# The Impact of Physical Activity on Adolescent Low Back Pain: A Systematic Review

**DOI:** 10.3390/jcm13195760

**Published:** 2024-09-27

**Authors:** Edoardo Costici, Sergio De Salvatore, Leonardo Oggiano, Sergio Sessa, Cloe Curri, Laura Ruzzini, Pier Francesco Costici

**Affiliations:** 1 Faculty of Medicine and Surgery, Saint Camillus International University of Health Sciences, 00131 Roma, Italy; 2 Orthopedic Unit, Department of Surgery, Bambino Gesù Children’s Hospital, 00165 Rome, Italy; leonardo.oggiano@opbg.net (L.O.); sergio.sessa@opbg.net (S.S.); cloe.curri@opbg.net (C.C.); laura.ruzzini@opbg.net (L.R.); pierfrancesco.costici@opbg.net (P.F.C.); 3 Research Unit of Orthopaedic and Trauma Surgery, Department of Medicine and Surgery, Università Campus Bio-Medico di Roma, Via Alvaro del Portillo 21, 00128 Roma, Italy

**Keywords:** low back pain, adolescent health, physical activity, sedentary behavior, prevention strategies, sport

## Abstract

**Background:** The relationship between physical activity and low back pain (LBP) in adolescents is complex, with conflicting evidence on whether activity is protective or a risk factor. The COVID-19 pandemic has introduced new challenges, increasing sedentary behaviors among adolescents. This systematic review updates the evidence on the association between physical activity and LBP in this population, focusing on the impact of the pandemic. **Methods:** A systematic search of PubMed, Cochrane Library, Web of Science, Medline, and SCOPUS identified observational studies published between January 2011 and December 2023. This review focused on adolescents aged 10 to 19 years, examining the effects of various physical activity levels and types on LBP incidence. Quality assessment was conducted using the ROBINS-I tool. **Results:** Twelve studies were included, with a total of 78,850 adolescents. The findings suggest a U-shaped relationship between physical activity and LBP, where low and high activity levels increase LBP risk, while moderate activity appears protective. The pandemic exacerbated LBP prevalence, likely due to increased sedentary behavior. Gender differences were noted, with females more likely to report LBP, particularly related to sports participation. **Conclusions:** Moderate physical activity may protect against LBP in adolescents, whereas both inactivity and excessive activity heighten risk. The pandemic’s impact highlights the need for balanced physical activity to prevent LBP. Further research should explore the long-term effects of these changes.

## 1. Introduction

Low back pain (LBP) is a significant public health concern worldwide, affecting individuals across various age groups, including adolescents [1,2]. While LBP has typically been viewed as a condition that mainly affects adults, recent studies have shown an alarming increase in its prevalence among younger populations [3] Epidemiological data indicate that LBP becomes more common as individuals age, with females being more susceptible than males [1]. However, the causes of LBP in adolescents are complex and not fully understood, involving a combination of biological, psychological, and social factors [4]. Adolescents are a unique demographic, experiencing rapid physical and psychological changes that may contribute to the development of musculoskeletal issues such as LBP [5]. Although physical activity is generally encouraged during this critical period due to its numerous health benefits, including promoting a healthy musculoskeletal system, cardiovascular fitness, and mental well-being, the relationship between physical activity and LBP in adolescents remains unclear [6,7,8].

The current literature presents conflicting evidence concerning the role of physical activity as a risk factor for LBP [9]. Some studies suggest that regular physical activity, particularly involving repetitive movements or high-impact sports, may contribute to the onset of LBP. This hypothesis is supported by research indicating that excessive or poorly managed physical activity could lead to overuse injuries, muscle imbalances, and mechanical stress on the spine, all of which are potential precursors to LBP. Conversely, other studies highlight the protective effects of moderate physical activity, suggesting that insufficient activity could lead to deconditioning, poor posture, and ultimately an increased risk of LBP [10,11]. Thus, the relationship between physical activity and LBP in adolescents appears to follow a U-shaped curve, where both low and high levels of activity are associated with higher LBP risk, while moderate levels may confer protection [12].

The most recent comprehensive systematic review addressing this topic included studies published until 2019, providing a comprehensive overview of the association between physical activity and LBP in adolescents at that time [13]. Since then, the field has seen the publication of new studies that expand on or challenge previous findings. Moreover, the advent of the COVID-19 pandemic has introduced new variables into the equation, significantly disrupting the daily lives of adolescents [14]. School closures, restrictions on sports and recreational activities, and increased screen time have led to more sedentary behaviors, which could have profound implications for adolescent musculoskeletal health, including the prevalence and severity of LBP [14]. 

For several reasons, understanding the interaction between physical activity and LBP in adolescents is crucial. First, adolescence is a pivotal time for establishing lifelong habits, including those related to physical activity [15]. Second, early-onset LBP has been shown to predict chronic pain and disability in adulthood, making prevention during adolescence particularly important [15]. Lastly, as public health strategies evolve in response to changing societal conditions, such as those brought about by the COVID-19 pandemic, it is imperative to base these strategies on the most current and comprehensive evidence available [16].

The purpose of this systematic review is to update the last systematic review by Kędra and colleagues [13] by reporting the current evidence on the impact of physical activity and LBP in adolescents aged 10 to 19 years. By providing an updated and nuanced understanding of this relationship, this review aims to identify potential gaps in the literature, offer recommendations for future research, and inform clinical practice and public health policies to mitigate the burden of LBP in this vulnerable population.

## 2. Materials and Methods

This systematic review followed PRISMA (preferred reporting items for systematic reviews and meta-analyses).

### 2.1. Eligibility Criteria

#### 2.1.1. Inclusion Criteria

- Study Design: only observational studies (prospective and retrospective cohort studies and cross-sectional studies) were included, as these designs are most appropriate for investigating associations and prevalence without introducing intervention-related biases.
- Population: The target population was adolescents aged 10 to 19 years, reflecting a period of significant growth and susceptibility to LBP. Studies were included if they involved adolescents with non-specific LBP, meaning LBP was not attributed to identifiable pathology (e.g., specific spinal disorders, infections, tumors).
- Exposure: Physical activity was assessed across various dimensions, including frequency, duration, intensity, and type (e.g., organized sports, recreational activities). Studies that objectively measured physical activity (e.g., using accelerometers) or relied on validated self-reported questionnaires were prioritized.
- Outcome: The primary outcome of interest was the presence or incidence of non-specific LBP. Studies were included if they provided clear operational definitions of LBP, with outcomes measured using validated tools (e.g., the Nordic musculoskeletal questionnaire).
- Language: to avoid translation bias and ensure accuracy, only studies published in English were considered.
- Publication Date: studies published between January 2011 and December 2023 were included, allowing for a comprehensive review of recent evidence, particularly studies conducted during and after the COVID-19 pandemic.

#### 2.1.2. Exclusion Criteria

1. Study Design: Experimental or interventional studies, and case-control designs, excluding non-observational studies. Non-observational studies were excluded because observational studies are most appropriate for investigating associations and prevalence without introducing intervention-related biases. These studies provide a better understanding of the natural history and risk factors associated with low back pain in adolescents, which aligns with the objectives of this review.
2. Population: Studies involving participants with specific causes of LBP (e.g., structural spinal deformities, trauma, systemic diseases) were excluded to eliminate confounding variables related to specific pathologies. Additionally, studies focusing exclusively on elite athletes were excluded due to the unique physical demands and risks associated with high-performance sports.
3. Outcomes: studies that did not clearly define LBP or relied on non-validated measures for physical activity or LBP were excluded to ensure data quality and comparability.

### 2.2. Information Sources

A comprehensive and systematic search of multiple electronic databases was conducted to identify relevant studies. The databases included PubMed, Cochrane Library, Web of Science, Medline, and SCOPUS. These databases were selected for their extensive coverage of biomedical, health, and sports sciences literature. Additionally, the grey literature was searched in OpenGrey, and conference proceedings were reviewed to capture any unpublished or ongoing studies that might have contributed to this review. Reference lists of included studies and relevant reviews were manually searched to ensure completeness. 

### 2.3. Search Strategy

The search strategy was carefully developed to capture relevant studies on the association between physical activity and low back pain (LBP) in adolescents. A combination of MeSH terms and free-text terms was used to ensure a comprehensive search across multiple databases. The search included terms related to “low back pain”, such as “back pain”, “lower back pain”, “lumbar pain”, and “non-specific low back pain”. These terms were combined with demographic terms like “child”, “adolescent”, “teen”, and “schoolchildren” to focus on the target population. Additionally, the search incorporated terms related to physical activity, including “youth sports”, “physical activities”, “activities of daily living”, “physical inactivity”, and “leisure activities”, to cover various aspects of physical activity. Physical activity was defined as any bodily movement produced by skeletal muscles that results in energy expenditure. This includes all forms of activity, from structured exercise and organized sports to daily activities such as walking, cycling, and household chores. Boolean operators were employed to refine the search and capture all relevant studies. The search was conducted systematically across selected databases, and the results were carefully screened for inclusion in this review. The search string is reported in Appendix A.

The search was conducted in December 2023 and the previous systematic review by Kędra et al. [13] was updated until 1 December 2019. Search results were imported into reference management software (CADIMA vers. 2.2.4.2—Apr 2023), and duplicates were identified and removed.

### 2.4. Study Selection

The study selection process was conducted in three stages: title screening, abstract screening, and full-text review.

1. Titles of all retrieved articles were independently screened by two reviewers (EC and SD). Studies were included at this stage if they mentioned low back pain (LBP) in adolescents and physical activity. Titles unrelated to these themes, such as those focusing on adult populations or specific spinal pathologies, were excluded. Ambiguous titles or those appearing to meet the inclusion criteria were retained for further review.
2. The two reviewers independently screened the abstracts of the selected articles. At this stage, the focus was on excluding studies that did not meet the inclusion criteria, such as those involving elite athletes, interventional studies, or studies lacking valid LBP or physical activity measures. Any discrepancies between the reviewers were resolved by discussion or, if necessary, by involving a third reviewer (CC). Studies that clearly defined the relationship between physical activity levels and LBP were included for full-text review.
3. Full-text versions of potentially eligible studies were obtained and assessed against predefined criteria. Only studies that provided clear operational definitions of non-specific LBP, utilized validated tools for measuring LBP and physical activity, and included adolescents aged 10–19 were included. A secondary manual search of reference lists from the selected articles was also conducted to identify any additional relevant studies. Studies not meeting these stricter criteria were excluded at this stage.

The entire study selection process was documented in a PRISMA flow diagram, detailing the number of studies screened, assessed for eligibility, and included in the review, along with reasons for exclusions at each stage. Cohen’s kappa was calculated and showed a score of 0.78, indicating substantial agreement between reviewers during the full-text screening phase.

### 2.5. Data Extraction

Data were extracted using a standardized form developed specifically for this review. A standardized data extraction form was developed specifically for this review to ensure consistency and accuracy during the data extraction process. This form underwent pilot testing on a small sample of studies (3 studies) to assess its adequacy and reliability. The pilot phase allowed us to identify ambiguities and challenges in extracting relevant data, which were subsequently addressed by making necessary adjustments to the form. Specifically, the reviewers identified some ambiguities in how exposure and outcome variables were categorized, which were subsequently addressed by refining the definitions of physical activity intensity and LBP prevalence.

The extracted data included general study information (such as the first author, year of publication, country of study, and journal) and specific details on study design, including the type of observational study, duration of follow-up for cohort studies, and sampling methods. Population characteristics were documented, capturing the age range, gender distribution, and relevant socio-demographic information. 

Regarding exposure, the type, intensity, frequency, and duration of physical activity were recorded, along with the methods used for assessment, such as questionnaires or objective measures like accelerometers. Outcome measures focused on the definition and measurement of LBP, including prevalence and incidence rates, and the duration of follow-up where applicable. The main results were documented, including risk estimates such as odds or hazard ratios, confidence intervals, and any subgroup analyses performed. 

Additional details on confounding factors were also extracted:

- Body mass index (BMI): some studies adjusted for BMI, recognizing its potential impact on physical activity levels and the likelihood of experiencing LBP.
- Gender: given the known gender differences in the prevalence of LBP, few studies performed subgroup analyses or controlled for gender in their statistical models.
- Socioeconomic status: a few studies took SES into account, acknowledging its influence on access to physical activities and healthcare, along with its potential relationship with musculoskeletal health.
- Previous injury or LBP history: few studies controlled for a history of LBP or previous injuries to better isolate the effect of physical activity on new cases of LBP.
- Physical activity type: some studies also addressed the type of physical activity (e.g., high-impact vs. low-impact sports), which may affect LBP risk differently.

Methodological quality indicators were noted based on the risk of bias assessment tool used in this review. Two reviewers (EC and SD) performed the data extraction to ensure accuracy and reliability, with any discrepancies resolved through consensus or by consulting a third reviewer (DC). All extracted data were systematically entered into a database for subsequent analysis. Efforts were made to address missing or incomplete data throughout the review process. In cases where key information was missing from the studies, the corresponding authors were contacted to seek clarification or obtain the missing data. For studies where this was not possible or no response was received, if key data (e.g., low back pain outcomes or physical activity levels) were absent, these studies were excluded from the final analysis to ensure the validity of our findings. Studies with missing data that would have affected the ability to conduct meaningful interpretation or analysis were excluded. All exclusions due to missing data were recorded and presented in the study’s PRISMA flow diagram to ensure transparency in the study selection process.

### 2.6. Quality Assessment

The methodological quality and risk of bias of the included studies were evaluated using the ROBINS-I (risk of bias in non-randomized studies of interventions) tool [17]. The ROBINS-I tool is specifically designed to assess bias in non-randomized studies of interventions, which often face unique challenges compared with randomized controlled trials. It evaluates bias across seven key domains: confounding, selection of participants, classification of interventions, deviations from intended interventions, missing data, measurement of outcomes, and selection of reported results. Each domain is critically examined to identify potential sources of bias that may affect the validity of the study’s findings. For confounding, which is particularly relevant in observational studies, the tool requires a detailed assessment of whether the most important confounding variables were identified, measured, and appropriately controlled for in the analysis. Common confounders in the included studies were factors such as patient age, sex, comorbidities, baseline physical activity levels, and the severity or chronicity of low back pain (LBP). To address these confounders, many studies applied multivariable regression models to adjust for confounding factors, while others used propensity score matching to balance comparison groups. However, the extent to which confounders were addressed varied across studies, with some providing only limited adjustment, potentially affecting the comparability of the results.

The ROBINS-I tool also evaluated potential biases arising from the non-random selection of participants, the methods used to measure physical activity and classify exposure status, and deviations from the intended interventions during the study period. Additionally, the tool assessed how missing data were handled and whether appropriate strategies, such as imputation, were employed to minimize bias.

While the ROBINS-I tool was deemed appropriate for this review, the tool has several limitations, including its complexity and potential subjectivity in certain domains. Alternatives such as the Newcastle–Ottawa scale (NOS) could be considered in future studies; however, ROBINS-I was chosen for its comprehensive assessment across several bias domains specific to non-randomized studies.

The measurement of outcomes for LBP was carefully evaluated to ensure reliability and validity, and any potential measurement biases were considered. The final domain of selective reporting was also examined, determining whether all prespecified outcomes were reported or if selective reporting was evident.

Each study was assigned an overall risk of bias rating, ranging from low to critical, based on the most severe domain rating identified. This evaluation was conducted independently by two reviewers to ensure objectivity, with any discrepancies resolved through discussion or by involving a third reviewer.

### 2.7. Data Synthesis

Given the heterogeneity of the included studies, a narrative synthesis was conducted. Studies were grouped according to their design, population characteristics, and the dimensions of physical activity measured. The synthesis focused on summarizing the associations between different levels and types of physical activity and LBP in adolescents, with attention to potential dose–response relationships and subgroup differences (e.g., by gender or specific types of activity).

Where possible, results were stratified by key confounders and effect modifiers. Meta-analysis was considered but ultimately not conducted due to significant heterogeneity in study methodologies and outcome measurements. Instead, the narrative synthesis aimed to identify patterns and gaps in the evidence, providing a comprehensive overview of the current state of knowledge.

The significant heterogeneity of the included studies drove the decision to conduct a narrative synthesis rather than a meta-analysis. Key differences that precluded meta-analysis included the following:

- Variability in study design: Included studies used a mix of observational designs, including cross-sectional and cohort studies. These designs had inherent methodological differences that made direct comparisons difficult, particularly in how exposures (physical activity) and outcomes (low back pain or LBP) were measured over time.
- Differences in population characteristics: Studies varied significantly in participant age range, geographic location, and demographic characteristics such as gender distribution and socioeconomic status. These differences resulted in variability in the external factors that influenced both physical activity levels and LBP outcomes, making it difficult to combine study results meaningfully.
- Inconsistency in outcome measures: Studies used different instruments and definitions to measure low back and physical activity. While some studies used validated questionnaires such as the Nordic musculoskeletal questionnaire, others relied on tools specifically developed for specific studies with different definitions of LBP and methods of measuring physical activity (e.g., self-reported data versus accelerometers). This variability in outcome measures further complicated the ability to synthesize the data quantitatively.
- Heterogeneity in types and intensity of physical activity: Included studies examined a wide range of physical activities, from structured sports to general physical activities of daily living, with varying levels of intensity. Some studies focused on high-impact sports, while others included low-impact or recreational activities. The different definitions and types of physical activity further complicated the efforts to conduct a meta-analysis.

Because of these significant differences in study design, population characteristics, and outcome measures, synthesis providing a narrative synthesis was deemed more appropriate. This approach allowed for a more flexible interpretation of the evidence by highlighting patterns and trends in the association between physical activity and LBP in adolescents while accounting for the inherent limitations and variability between studies.

## 3. Results

### 3.1. Study Selection

**Figure 1:**
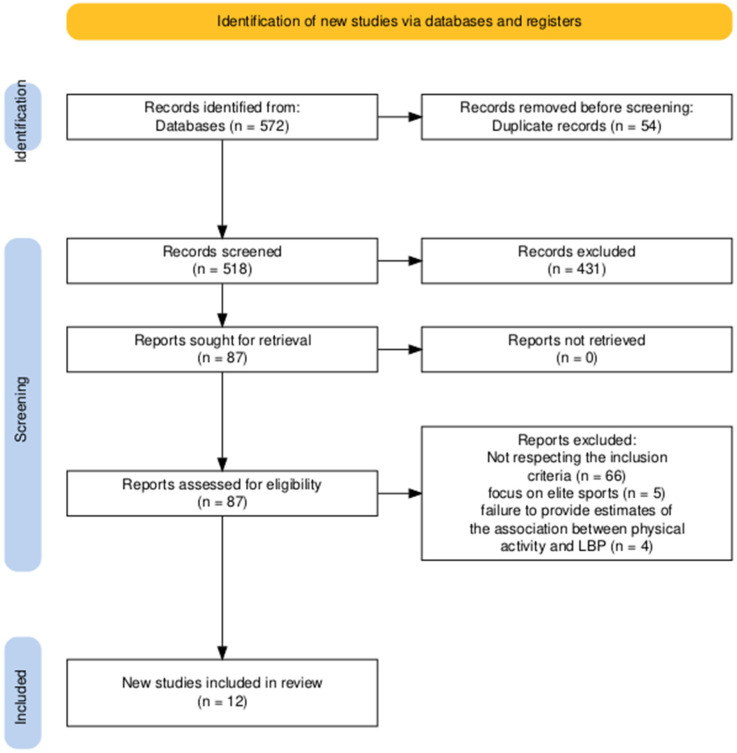
Prisma checklist.

The initial database search identified 572 articles. After removing 54 duplicates, 518 articles remained. The titles and abstracts of these articles were screened, resulting in the exclusion of 431 articles that did not meet the inclusion criteria. The full texts of the remaining 87 articles were assessed for eligibility. Of these, 75 articles were excluded for the following reasons: lack of information on physical activity (7 articles), focus on elite sports (5 articles), and failure to provide estimates of the association between physical activity and LBP (4 articles). Consequently, 12 studies met the inclusion criteria and were included in the final analysis. The PRISMA flow diagram illustrates the study selection process (Figure 1).

### 3.2. Study Characteristics

The 12-included studies comprised a total sample of 78,850 adolescents from eight different countries. The studies varied in design, with five employing cohort designs and seven utilizing cross-sectional designs. This variation allowed for a comprehensive examination of the association between physical activity and LBP across different contexts and time frames.

Regarding LBP measurement, four studies employed the standardized Nordic questionnaire, a widely recognized tool for assessing musculoskeletal symptoms, including LBP. The remaining eight studies used questionnaires specifically developed for their respective studies. These instruments varied in their approach, with some focusing on specific aspects of LBP, such as frequency and intensity, while others took a more general approach.

Physical activity was assessed in various ways across the studies. Two studies measured the weekly duration of physical activity, quantitatively evaluating how much time adolescents spent engaging in physical activities. Five studies focused on the intensity of physical activity, assessing the level of effort or energy expenditure associated with these activities. The remaining five studies examined participation in sports outside of school hours, considering both organized sports and informal recreational activities. Data collection methods included multiple-choice questionnaires, interviews, and in some cases, objective measures such as accelerometers, though these were less commonly used.

A detailed summary of the characteristics of the included studies is presented in Table 1, which outlines critical aspects such as study design, population demographics, physical activity and LBP assessment methods, and the main findings of each study.

### 3.3. Risk of Bias in Studies

The risk of bias across the included studies was assessed using the ROBINS-I tool. The studies varied in methodological rigor, with five studies classified as having a low risk of bias and seven categorized as having a moderate risk of bias. The primary sources of bias identified were related to confounding and participant selection.

Confounding was a concern in several studies, particularly those that did not adequately control for potential confounders as body mass index (BMI), socioeconomic status, or previous history of LBP. Bias due to participant selection was also notable, especially in studies where the sampling methods may not have ensured the representativeness of the adolescent population.

**Figure 2:**
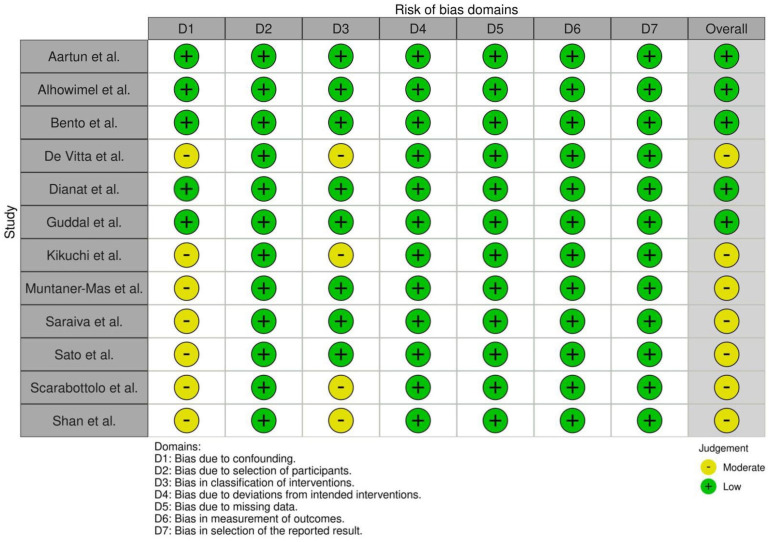
Risk of bias assessment using ROBINS tool [5,9,10,11,12,18,19,20,21,22,23,24].

Figure 2 summarizes the risk of bias assessment for each study, highlighting the areas where potential biases were most likely to influence the findings.

### 3.4. Results of the Individual Studies

The heterogeneity of the included studies in terms of exposure (physical activity) and outcome measures precluded a meta-analytic approach. Instead, a qualitative synthesis was conducted, identifying key patterns and relationships across the studies.

Six studies reported a positive association between physical activity and LBP, indicating that very low and very high levels of physical activity are associated with an increased risk of LBP. This finding suggests a U-shaped relationship, where moderate physical activity levels may offer a protective effect, while extremes in either direction elevate the risk of LBP.

Three studies found that moderate physical activity levels were associated with a reduced risk of LBP in adolescents.

Three studies reported no significant association between physical activity and LBP. These findings highlight the complexity of the relationship and suggest that other factors, such as the type of physical activity, individual biomechanics, or environmental influences, might play a role in determining LBP risk.

### 3.5. Subgroup Analyses

Subgroup analyses were conducted to explore potential differences in the association between physical activity and LBP based on gender and the type of physical activity.

#### 3.5.1. Gender Differences

Several studies identified gender differences in the prevalence and risk factors for LBP; specifically, girls who participated in sports were more likely to report LBP compared with boys. This finding aligns with broader epidemiological data suggesting a higher lifetime prevalence of back pain in females. Possible explanations include hormonal differences, variations in muscle strength, and differences in sports participation patterns between genders.

#### 3.5.2. Type of Physical Activity

The studies varied in their assessment of different types of physical activity. The heterogeneity in how physical activities were categorized and measured across studies made it difficult to draw definitive conclusions about specific sports or activities.

### 3.6. Certainty of Evidence

Overall, the evidence suggests that moderate physical activity is protective against LBP in adolescents, while both sedentary behavior and high levels of physical activity increase the risk. However, the certainty of this evidence is moderate, given the methodological limitations of the included studies, such as reliance on self-reported data and variability in how physical activity and LBP were defined and measured. The findings underscore the importance of promoting balanced physical activity levels among adolescents to maintain back health.

## 4. Discussion

### 4.1. Main Findings

The complex relationship between physical activity (PA) and low back pain (LBP) in adolescents was extensively analyzed. A consistent pattern emerged, indicating a U-shaped relationship between PA levels and the risk of LBP. Both low and high levels of physical activity were associated with an increased risk of LBP, whereas moderate levels of activity appeared to exert a protective effect. 

### 4.2. Result Analysis

This systematic review aimed to critically assess the relationship between PA and LBP in adolescents, with a particular emphasis on the impact of the COVID-19 pandemic. By synthesizing data from 12 studies involving a diverse cohort of 78,850 adolescents across eight countries, this review provides a comprehensive understanding of how varying levels of physical activity influence the risk of LBP in this vulnerable population.

The last relevant systematic review that examined the relationship between physical activity and LBP in adolescents included studies up to 2019. That review provided a solid foundation for understanding the dynamics of PA and LBP; however, it did not account for the dramatic lifestyle changes brought about by the COVID-19 pandemic. The current review, which includes studies published during the COVID-19 era, offers a unique opportunity to compare pre-pandemic and pandemic-era evidence. This comparison is crucial for understanding how increased sedentary behaviors, driven by lockdowns, school closures, and restricted access to recreational activities, have influenced the prevalence and severity of LBP in adolescents. 

The findings of this review suggest that the pandemic has exacerbated LBP incidence among adolescents, likely due to prolonged screen time and poor posture associated with increased digital device use, as highlighted in studies like Mokhtarinia et al. [14].

The findings underscore a U-shaped relationship between physical activity levels and LBP risk, where very low and very high activity levels are associated with increased LBP incidence. In contrast, moderate levels appear to be protective. This pattern aligns with the broader body of the literature on musculoskeletal health, particularly the work by Heneweer et al. [25], which proposed that insufficient and excessive physical activity can contribute to back problems. Similarly, the review by Sitthipornvorakul et al. [26] highlighted the need for a balanced approach to physical activity, noting that moderate exercise promotes spinal health, whereas extremes in activity levels may lead to musculoskeletal strain and pain.

Gender differences were also prominent in this review, with females more likely to report LBP than males, particularly those involved in sports activities [27]. This observation is in line with the findings of Kikuchi et al. [10,11], who reported that female adolescents had a higher prevalence of LBP associated with sports participation. These gender disparities may be attributed to several factors, including hormonal differences, anatomical variations, and different patterns of physical activity engagement between males and females [10,11]. This highlights the importance of tailoring interventions to address gender-specific risks in the prevention and management of LBP in adolescents.

Moreover, this review underscores the need to distinguish between different types of physical activity when assessing their impact on LBP. While general physical activity benefits overall health, specific activities requiring repetitive strain or heavy lifting may increase the risk of LBP. For example, sports emphasizing core strength and flexibility, like yoga or swimming [28,29], may protect against LBP, whereas high-impact activities like weightlifting or contact sports may exacerbate the condition. This distinction is critical for designing effective prevention strategies and was highlighted in studies like those by Muntaner-Mas et al. [12] and Zemková [30] that explored the differential impacts of various physical activities on adolescent spinal health.

### 4.3. Limitations

Despite the strengths of this systematic review, several limitations must be acknowledged. The heterogeneity in study design, particularly in measuring physical activity and LBP, presents data synthesis and interpretation challenges. Most of the included studies relied on self-reported questionnaires to assess physical activity and LBP, which introduces potential biases such as recall and social desirability. These biases may have influenced the accuracy of the reported associations. Furthermore, the predominance of cross-sectional designs among the included studies limits the ability to establish causality between physical activity levels and LBP. Studies that utilized objective measures (such as accelerometers to assess physical activity) should be considered more accurate to address the reliance on self-reported data, as they provided more reliable and accurate data than self-reported questionnaires.

Longitudinal studies would be necessary to understand better the temporal dynamics and potential causal mechanisms underlying this relationship. Another limitation is the variability in how different types of physical activity were categorized across studies. This variability complicates the identification of specific activities that might be particularly beneficial or harmful to adolescent back health. Additionally, the impact of other possible confounding variables such as body weight [31], spinal abnormalities [32] and socioeconomic status [33] should also be considered in studies. These factors could potentially influence physical activity patterns and LBP risk, suggesting that future research should incorporate more comprehensive control for these variables to isolate physical activity’s effects on LBP.

### 4.4. Recommendations

Healthcare providers, educators, and athletic trainers must place great emphasis on encouraging youth to engage in regular physical activity that is neither too minimal nor too excessive. Finding the right level of physical activity is important for long-term back health. Promoting and maintaining moderate and balanced activity should be the top priority. Additionally, the development of well-structured targeted interventions that target both physical and mental well-being of adolescents will be needed in the post-pandemic era, when changes in lifestyle and behavior require a renewed focus on health that will be of the utmost importance. This proactive approach will be critical to protecting adolescents’ back health at a time of rapid change and new challenges.

### 4.5. Future Research

Future research should focus on overcoming the limitations inherent in the reliance on self-reported data by using objective measures of physical activity, such as accelerometers or wearable fitness trackers, and validated tools for assessing LBP. Additionally, the following suggestions are recommended:

- Longitudinal studies: There is a clear need for longitudinal study designs that can track changes in physical activity and LBP over time. Such studies would help establish a causal relationship between physical activity levels and developing or preventing LBP in adolescents. Longitudinal data would also enable researchers to observe the long-term effects of physical activity patterns from adolescence into adulthood.
- Larger and diverse populations: Future research should include larger more diverse populations, particularly in terms of geography, gender, and socioeconomic status, to enhance the generalizability of the findings. Studies should aim to recruit representative samples to capture variations across different demographic groups, which would help in understanding how specific subgroups are affected differently by physical activity in relation to LBP.
- Standardized outcome measures: Future research should prioritize the use of standardized and validated outcome measures for both physical activity and LBP to allow for more meaningful comparisons across studies and improve the reliability of meta-analyses. This would also help in addressing the heterogeneity observed in this review.

## 5. Conclusions

This systematic review provides valuable insights into the complex relationship between physical activity and low back pain (LBP) in adolescents. The evidence suggests that moderate levels of physical activity are protective against LBP, while both low and high levels of activity increase the risk. In terms of future research, the following areas should be prioritized: longitudinal studies including more diverse population samples and the use of standardized measures for outcome. Healthcare providers should encourage adolescents to engage in regular but not excessive physical activity, and developing targeted interventions will be vital for protecting adolescent back health in the evolving post-pandemic landscape.

## Figures and Tables

**Table 1:** Study characteristics and findings.

Study	Year	Study Type	Country	Age (Range)	Sample Size (Female)	Outcome Summary	Measurement of Physical Activity	Physical Activity	Main Findings
Aartun et al. [18]	2016	CS	Denmark	12 (11–13)	144 (60)	LBP presentation (often, sometimes, once or twice)	GT3X triaxial accelerometer	Physical activity level (sedentary, light, moderate, vigorous)	No direct relation between LBP and physical activity; adolescents who reported vigorous physical activity in the questionnaire are more affected by LBP
Alhowimel et al. [19]	2022	CSS	Saudi Arabia	16 (14–18)	2000 (722)	Closed-ended question (self reported Arabic questionnaire)	Questionnaire	(1) less than 1 h/week (2) 2–3 h/week (3) 3–4 h/week (4) more than 4 h/week (5) never	Physical activity is reported as a risk factors in development of LBP
Bento et al. [20]	2019	CSS	Brazil	16.5 (15–18)	1628 (830)	Nordic questionnaire + LBP in past years	Baecke questionnaire	Self-declared level of physical activity (very active, sufficiently active, insufficiently active)	There was no association between low back pain and physical activity
De Vitta et al. [21]	2021	CS	Brazil	16 (14–18)	757 (332)	Nordic questionnaire	Baecke questionnaire	3 levels of physical activity level (active, moderately active, and sedentary)	Among adolescents reporting low back pain (LBP), 24.5% have a sedentary lifestyle, 51% are moderately active, while only 24.5% are active
Dianat et al. [22]	2017	CSS	Iran	16 (13–19)	1611 (860)	Questionnaire	Questionnaire	(1) Less than 1 h/week, (2) 1–3 h/week, (3) >3 h/week	No relation between LBP and physical activity
Guddal et al. [5]	2017	CSS	Norway	16 (13–19)	7596 (3831)	Questionnaire	HBSC questionnaire	3 levels of physical activity level (low activity, moderate activity, and high activity)	In comparison with a low level of physical activity, a moderate physical activity level showed a significant association with reduced odds of experiencing LBP in both girls and boys
Kikuchi et al. [10]	2019	CS	Japan	12 (9–15)	31,419 (6052)	Questionnaire	Questionnaire	Sport outside school	LBP more prevalent in adolescents who participate in sports than in others
Muntaner-Mas et al. [12]	2018	CSS	Spain	11 (10–12)	2032 (943)	Questionnaire	Questionnaire	Sport outside school	Participants who spend more than four hours per week are more likely to have LBP
Saraiva et al. [9]	2019	CS	Brazil	13.5 (10–17)	870 (488)	Nordic questionnaire	Questionnaire	Continuity of physical activity (yes or no)	Adolescents who consistently engaged in physical activity from childhood through adolescence had a reduced risk of developing back pain compared with those who did not sustain their physical activity levels
Sato et al. [11]	2011	CSS	Japan	12 (9–15)	26,766 (12,430)	Questionnaire	Questionnaire	Physical activity outside school	Sport activity increases the risk of low back pain
Scarabottolo et al. [23]	2017	CSS	Brazil	13.5 (10–17)	1011 (557)	Nordic questionnaire	Baecke questionnaire	Total physical activity level	An association between sedentary behavior and low back pain was observed only in girls
Shan et al. [24]	2013	CS	China	17 (15–19)	3016 (1556)	Questionnaire	Questionnaire	Intensity of physical activity, frequency of weekly exercise, and average time of each exercise	Physical activity is generally considered a protective factor against musculoskeletal diseases

CS—cohort study; CSS—cross-sectional study; LBP—low back pain.

## Data Availability

The datasets used and/or analyzed during the current study are not publicly available due to our policy statement of sharing clinical data only on request but are available from the corresponding author on reasonable request.

## Appendix A. Search String

PubMed:

((“Low back pain”[MeSH]) OR (“Back pain”[Title/Abstract] OR “low back pain”[Title/Abstract] OR “lower back pain”[Title/Abstract] OR “lumbar pain”[Title/Abstract] OR “non-specific low back pain”[Title/Abstract] OR “lumbosacral pain”[Title/Abstract])) AND ((“Child”[MeSH]) OR “Adolescent”[MeSH] OR Child*[Text Word] OR adolescent*[Text Word] OR teen*[Text Word] OR schoolchildren[Text Word] OR “school children”[Text Word]) AND ((“Youth Sports”[MeSH]) OR “Physical activity”[Text Word] OR “physical activities”[Text Word] OR “activities of daily living”[Text Word] OR “physical inactivity”[Text Word] OR “leisure activities”[Text Word] OR “level of physical activity”[Text Word] OR sport*[Text Word]).

The Cochrane Library:

([Low back pain] OR [Back pain] OR [low back pain] OR [lower back pain] OR [lumbar pain] OR [non-specific low back pain] OR [lumbosacral pain]) AND ([Child] OR [Adolescent] OR [Child*] OR [adolescent*] OR [teen*] OR [13] OR [“school children”]) AND ([Youth Sports] OR [Physical activity] OR [physical activities] OR [activities of daily living] OR [physical inactivity] OR [level of physical activity] OR [sport*]).

Web of Science:

TS = (“Low back pain” OR “Back pain” OR “low back pain” OR “lower back pain” OR “lumbar pain” OR “non-specific low back pain” OR “lumbosacral pain”) AND TS = (“Child” OR “Adolescent” OR Child* OR adolescent* OR teen* OR schoolchildren OR “school children”) AND TS = (“Youth Sports” OR “Physical activity” OR “physical activities” OR “activities of daily living” OR “physical inactivity” OR “leisure activities” OR “level of physical activity” OR sport*).

Medline:

((“Low back pain”[MeSH]) OR (“Back pain”[Title/Abstract] OR “low back pain”[Title/Abstract] OR “lower back pain”[Title/Abstract] OR “lumbar pain”[Title/Abstract] OR “non-specific low back pain”[Title/Abstract] OR “lumbosacral pain”[Title/Abstract])) AND ((“Child”[MeSH]) OR “Adolescent”[MeSH] OR Child*[Text Word] OR adolescent*[Text Word] OR teen*[Text Word] OR schoolchildren[Text Word] OR “school children”[Text Word]) AND ((“Youth Sports”[MeSH]) OR “Physical activity”[Text Word] OR “physical activities”[Text Word] OR “activities of daily living”[Text Word] OR “physical inactivity”[Text Word] OR “leisure activities”[Text Word] OR “level of physical activity”[Text Word] OR sport*[Text Word]).

SCOPUS:

TITLE-ABS-KEY(“Low back pain” OR “Back pain” OR “low back pain” OR “lower back pain” OR “lumbar pain” OR “non-specific low back pain” OR “lumbosacral pain”) AND TITLE-ABS-KEY(“Child” OR “Adolescent” OR Child* OR adolescent* OR teen* OR schoolchildren OR “school children”) AND TITLE-ABS-KEY(“Youth Sports” OR “Physical activity” OR “physical activities” OR “activities of daily living” OR “physical inactivity” OR “leisure activities” OR “level of physical activity” OR sport*).
